# Impact of Surgical Treatment on the Quality of Life in Patients With Chronic Otitis Media With Cholesteatoma: A Prospective Study

**DOI:** 10.7759/cureus.99384

**Published:** 2025-12-16

**Authors:** Jocelyne García-Vela, Jose Treviño-González, Andrea Citlalli Flores-Álvarez

**Affiliations:** 1 Otolaryngology - Head and Neck Surgery, “Dr. José Eleuterio González” University Hospital, Monterrey, MEX

**Keywords:** cholesteatoma, chronic otitis media, comq-12, endoscopic tympanoplasty, hearing outcomes, hrqol, mastoidectomy, otologic surgery, quality of life

## Abstract

Introduction

Chronic otitis media with cholesteatoma (COMC) is a progressive and potentially destructive middle ear disease, with surgery as the treatment of choice. Its auditory, emotional, and social consequences significantly affect patients’ quality of life. The Chronic Otitis Media Questionnaire-12, Mexican version (COMQ-12-Mx), allows a validated assessment of this impact in the Mexican population.

Objective

The main objective of this study is to evaluate the effect of surgical treatment on quality of life and auditory function in patients with COMC, using the COMQ-12-Mx.

Material and methods

A prospective cohort study was conducted at the University Center of Otolaryngology and Head and Neck Surgery, “Dr. José Eleuterio González” University Hospital, Monterrey, Mexico, from May to October 2025. Twenty-five adults with clinically and radiologically confirmed COMC were included. The COMQ-12-Mx and audiometric tests were applied before and three months after surgery.

Results

Twenty-five affected ears were analyzed (median age 47 years; 56% female). The most frequent comorbidities were type 2 diabetes (32%), bilateral chronic otitis media (28%), and hypertension (16%). Main symptoms included tinnitus (68%), otalgia (64%), and otorrhea (64%). The attic-antral region was most commonly involved (68%). Simple mastoidectomy (76%) and type III tympanoplasty (78.6%) were the predominant techniques. Postoperative complications occurred in 24%, mainly graft perforation (16%). The pure-tone average (PTA) showed a trend toward improvement (48 dB preoperative vs 42 dB postoperative; p = 0.059). Total COMQ-12-Mx scores significantly decreased (29 vs 21; p < 0.001), with improvement across all subdomains and a large effect size (r = 0.87). Both canal wall up (CWU) and endoscopic techniques improved global scores, while only CWU showed significant PTA improvement (p = 0.041).

Conclusions

Surgery for COMC was associated with within-group improvement in quality of life, particularly in symptoms and emotional impact. The COMQ-12-Mx adequately detected postoperative changes and served as a reliable tool to assess clinical and functional outcomes after surgical treatment.

## Introduction

Chronic otitis media with cholesteatoma (COMC) is a progressive and potentially destructive middle ear disease characterized by the abnormal proliferation of squamous epithelium, which can erode bony structures and lead to severe complications if not treated promptly [1,2]. Its main consequences include conductive hearing loss due to ossicular chain destruction and progressive erosion of the temporal bone, most commonly involving the mastoid air cells, the scutum, and the bony walls of the epitympanum and antrum. In advanced cases, the disease may also extend to intracranial structures. COMC remains a prevalent condition and significant public health concern despite scientific progress [3]. Its prevalence ranges from 0.4% to 4.8%, depending on the studied population and diagnostic criteria, with a higher incidence observed in regions with limited access to medical care. Factors such as recurrent middle ear infections during childhood, Eustachian tube dysfunction, and unfavorable socioeconomic conditions increase the risk of developing the disease [4-6].

Surgical intervention, typically through mastoidectomy with or without tympanoplasty, is the treatment of choice, aiming to eradicate the cholesteatoma, prevent recurrence, and improve hearing function. However, disease recurrence and postoperative functional sequelae - such as persistent conductive or mixed hearing loss, recurrent otorrhea, instability of the reconstructed middle ear space, or the need for revision surgery - remain significant clinical challenges [5,7,8]. Given its auditory, emotional, and social impact, COMC significantly affects patients’ quality of life. Instruments such as the Chronic Otitis Media Questionnaire-12 (COMQ-12) have proven useful in assessing this impact and guiding more effective therapeutic strategies [9-13].

This study aimed to evaluate the impact of surgical treatment on the quality of life of patients with COMC, contributing to a better understanding of its clinical and functional outcomes.

## Materials and methods

Study design and setting

We conducted an observational, analytical, prospective, longitudinal cohort study including adult patients (≥18 years) with a clinical and radiological diagnosis of COMC. All patients were consecutively recruited and evaluated between May and October 2025 at the Department of Otolaryngology and Head and Neck Surgery of “Dr. José Eleuterio González” University Hospital, Monterrey, Mexico. Follow-up consisted of a standardized postoperative evaluation at three months, during which a repeat COMQ-12-Mx (Mexican version) questionnaire and audiometric assessments were performed. All patients completed follow-up.

Exclusion criteria included a history of previous mastoidectomy on the affected ear - since prior surgery reflects recurrent or residual cholesteatoma and alters the anatomical baseline - being younger than 18 years, having incomplete clinical or radiological information necessary for diagnosis or outcome assessment, or being unable to complete the COMQ-12-Mx questionnaire. Five patients met these criteria: two with a history of mastoidectomy, and three who were under 18 years of age.

Study size calculation

The sample size was estimated to detect a mean difference in COMQ-12-Mx scores between preoperative and postoperative assessments (paired design). Based on the values reported by Schouwenaar et al. (mean reduction from 24.91 to 14.8) [12] and assuming a significance level of α = 0.05 and 90% power (β = 0.10), a minimum of 21 participants was required. An additional 10% was added to account for potential losses to follow-up, resulting in a recruitment target of 23 participants. Ultimately, a total of 25 patients were included, exceeding the minimum sample required for the primary outcome.

Demographic and clinical data

Demographic variables (age and sex), body mass index (BMI), comorbidities, risk factors, and pre-treatment laboratory data were collected. Abnormal C-reactive protein (CRP) values were defined according to the hospital laboratory reference range (>10 mg/L). A structured survey was conducted among all participants to identify the most common auditory symptoms. A complete otolaryngological examination was performed by the same otolaryngologist in all cases to ensure consistency. All surgeries were performed by the same senior surgeon as inpatient procedures. All patients underwent preoperative pure-tone audiometry and computed tomography (CT). The surgical technique was determined pre- and intraoperatively, according to disease extension, anatomic configuration, and the presence of complications. 

Audiological evaluation

Audiological assessment was performed in all participants by a certified audiologist. Conventional pure-tone audiometry (0.5, 0.75, 1, 2, 4, and 8 kHz), tympanometry, stapedial reflexes, and speech audiometry were included. Tests were conducted within a soundproof booth using a calibrated Interacoustics AC40 audiometer (Interacoustics A/S, Middelfart, Denmark) in accordance with ANSI/ASA S3.6-2004 standards. Each frequency was manually verified in 5 dB increments. Air conduction (AC) and bone conduction (BC) thresholds were averaged at 0.5, 1, 2, and 4 kHz to calculate the pure-tone average (PTA) for each ear. Hearing loss was defined as an average threshold elevation >20 dB HL across the tested frequencies and classified according to the World Health Organization (WHO) 2021 Hearing Grading System, based on the affected-ear PTA [14].

Quality of life assessment

Health-related quality of life was assessed using the COMQ-12-Mx questionnaire, adapted and validated for the Mexican population by Celis-Aguilar et al. [9]. Permission to use the COMQ-12-Mx was obtained from the corresponding author. This COMQ-12, developed by Phillips et al. [15], was structured into four subdomains to assess the impact of chronic otitis media (COM): symptom severity (sum of items 1-7), lifestyle and work impact (items 8 and 9), health services impact (items 10 and 11), and emotional distress (item 12). For the present analysis, the last subdomain (general impact) was renamed emotional distress to better reflect the psychological aspects assessed. Each item is rated on a six-point Likert scale (0 = no impact or sporadic occurrence; 5 = most severe impact or daily occurrence), yielding a total score ranging from 0 to 60. 

Statistical analysis

Quantitative variables were analyzed as continuous data. Normality was assessed using the Shapiro-Wilk test, skewness, and kurtosis, demonstrating non-normal distributions; therefore, continuous variables were summarized as median and interquartile range (IQR) and analyzed with non-parametric methods. No continuous variables were categorized; the WHO audiometric grades, COMQ-12-Mx subdimensions, and surgical technique groups reflect standardized clinical classifications. 

Paired analyses (pre- vs postoperative COMQ-12-Mx total and subdimension scores; pre- vs postoperative audiometric thresholds; item-level effect sizes; and pre- vs postoperative comparisons within each surgical technique) were performed using the Wilcoxon signed-rank test.

Independent-group analyses (comparison of preoperative COMQ-12-Mx total and subdimension scores according to clinical presentation) were conducted using the Mann-Whitney U test.

The within-technique analyses were considered exploratory, given the limited sample size in each subgroup. The canal wall down (CWD) subgroup was excluded from these analyses due to its very small sample (n = 2). Accordingly, no formal adjustment for multiple comparisons was applied. Due to the single-group design and limited sample size, no multivariable or confounding-control methods were applied. All patients completed follow-up, and no missing data were present. A p-value < 0.05 was considered statistically significant. All analyses were performed using IBM SPSS Statistics for Windows, Version 30 (Released 2024; IBM Corp., Armonk, NY, USA).

Ethical disclosure

The study was performed in accordance with the ethical standards laid down in the 1964 Declaration of Helsinki and its later amendments. This protocol was approved by the Ethics Committee in Research of the “Dr. José Eleuterio González” University Hospital, Universidad Autónoma de Nuevo León under the number 25-00001. The surgical procedures were part of routine clinical management and were not influenced by the study. Written informed consent was waived by the ethics committee, and verbal informed consent was obtained from all participants before completing the questionnaire. All data were anonymized prior to analysis.

## Results

Demographic and clinical features

A total of 25 patients were evaluated. The median age was 47 years (32.5-56.5), 42 years (24-47) for men, and 51.5 years (38.5-59.25) for women, with a male-to-female ratio of 1:1.27. The median BMI was 27 kg/m² (22.5-30). Most participants had completed high school education (44%). The most frequent comorbidities were type 2 diabetes mellitus (32%) and bilateral COM (16%).

The median duration of symptoms before diagnosis was two years (0.45-3.5), and the median time since the initial diagnosis was four years (1-10). The most common presenting symptoms were tinnitus (68%), otorrhea (64%), and otalgia (64%). No significant differences were found in preoperative COMQ-12-Mx total or subdimension scores between patients with and without otorrhea (p > 0.05). Similarly, no significant differences were observed for the presence of external auditory canal edema, retroauricular edema, or tinnitus. A detailed summary of baseline clinical characteristics and laboratory values is presented in Table 1.

**Table 1:** Participant characteristics (N = 25) Data are presented as median (IQR) and number (percentage). COM: chronic otitis media; BMI: body mass index; EAC: external auditory canal; CRP: C-reactive protein; IQR, interquartile range

Factors	Results
Age (years)	47 (32.5 - 56.5)
Men	11 (44%)
Women	14 (56%)
BMI	27 (22.5 - 30)
Level of education	
None	1 (4%)
Primary school	4 (16%)
Secondary school	2 (8%)
High school	11 (44%)
Professional career	5 (20%)
Postgraduate education	2 (8%)
Risk factors	
Alcoholism	6 (24%)
Smoking	3 (12%)
Pack-year	8.5 (5 - 12)
Drugs	6 (24%)
Comorbidities	
Type 2 diabetes mellitus	8 (32%)
Bilateral COM	4 (16%)
Arterial hypertension	4 (16%)
Obesity	3 (12%)
Hypothyroidism	2 (8%)
Malignant neoplasm	2 (8%)
Melanoma	1 (4%)
Adenocarcinoma	1 (4%)
Rheumatoid arthritis	1 (4%)
Chronic kidney disease	1 (4%)
Hepatitis C	1 (4%)
Hepatic steatosis	1 (4%)
History of ventilation tube placement	4 (16%)
Number of previous ventilation tube placements (per patient)	1.5 (1 - 2)
Clinical presentation	
Duration from symptom onset to diagnosis (years)	2 (0.45 - 3.5)
Tinnitus	17 (68%)
Otalgia	16 (64%)
Otorrhea	16 (64%)
EAC edema	7 (28%)
Retroauricular edema	2 (8%)
Pre-treatment laboratory data	
Abnormal CRP	2 (8%)
CRP, mg/dL	13.5 (1 - 26)
Leukocytes, cells/μL	6.85 (6.45 - 7.9)

Radiological findings

All patients underwent preoperative CT, which enabled a detailed evaluation of disease extension and localization. The attic-antrum region was the most frequently affected site (68%), followed by the attic/epitympanum (32%), mastoid air cells (32%), mesotympanum (28%), endaural extrusion (12%), additus ad antrum (4%), and middle ear (4%). The distribution of involvement was symmetrical between sides (right ear: 48%; left ear: 52%).

Therapeutic approaches

Prior to surgery, 36% of patients had received antimicrobial therapy, most commonly ciprofloxacin monotherapy (20%), while others were treated with combined antibiotic regimens (4%) or an unspecified antibiotic (12%). All patients subsequently underwent surgical management. The most frequently performed procedure was simple mastoidectomy (76%), of which the majority were carried out with preservation of the posterior canal wall up (CWU: 89.4%). Additionally, 56% of the patients required tympanoplasty, most commonly type III (78.5%).

Furthermore, surgical techniques aimed at managing the epitympanum were employed, including attic-antrum exclusion, atticotomy, antrum exclusion-attic exposure, and attic-antrum exposure. Postoperative complications occurred in 24% of patients, the most frequent being perforation or graft failure of the neotympanum (16%) (Table 2).

**Table 2:** Therapeutic approaches and postoperative complications (N = 25) Percentages for mastoidectomy types were calculated based on the patients who underwent mastoidectomy (n = 19); percentages for tympanoplasty types were calculated based on the patients who underwent tympanoplasty (n = 14); percentages for endoscopic subprocedures were calculated based on the patients who underwent endoscopic resection (n = 4). Data are presented as number (percentage). CWU, canal wall up; CWD, canal wall down

Factors	Results
Previous antimicrobial therapy	9 (36%)
Ciprofloxacin monotherapy	5 (20%)
Combination antibiotic therapy	1 (4%)
Unknown/not reported antibiotic	3 (12%)
Surgical intervention	
Retrograde tympanomastoidectomy	2 (8%)
Simple mastoidectomy	19 (76%)
CWU mastoidectomy	17 (89.4%)
CWD mastoidectomy	2 (10.5%)
Tympanoplasty	14 (56%)
Type II	2 (14.2%)
Type III	11 (78.5%)
Type IV	1 (7.1%)
Endoscopic resection	4 (16%)
Atticotomy	1 (25%)
Attic exposure	1 (25%)
Antrum exclusion	1 (25%)
Attic-exclusion antrum exposure	1 (25%)
Ventilation tube placement	4 (16%)
Turbinate resection	1 (4%)
Surgical complication	6 (24%)
Neotympanum perforation	4 (16%)
Graft failure	1 (4%)
Graft retraction	1 (4%)
Recurrence	1 (4%)

Hearing outcome 

In the audiometric comparison, the PTA showed a trend toward improvement, although the difference did not reach statistical significance (preoperative 48 (43.5-80) vs postoperative 42 (34.5-79), p = 0.059) (Table 3). A change was observed in the distribution of the audiometric classification, with an increase in the proportion of patients presenting normal hearing (0-2) and mild hearing loss (5-9), although the proportion of those with severe hearing loss also increased (3-8).

**Table 3:** Comparison of pre and postoperative audiometric outcomes and COMQ-12-Mx scores (N = 25) Data are presented as median (IQR) and number (percentage). ^a^p-value has been calculated using the Wilcoxon signed-rank test. A p-value < 0.05 was considered statistically significant. PTA, pure-tone average; IQR, interquartile range; COMQ-12-Mx, Chronic Otitis Media Questionnaire-12, Mexican version

Factors	Assessment timepoint		Test value	p-value^a^
	Preoperative (N = 25)	Postoperative (N = 25)		
PTA (dB)	48 (43.5 - 80)	42 (34.5 - 79)	-1.889	0.059
Audiometric classification				
Normal hearing	0 (0%)	2 (8%)	-	-
Mild hearing loss	5 (20%)	9 (36%)	-	-
Moderate hearing loss	10 (40%)	4 (16%)	-	-
Moderately severe hearing loss	3 (12%)	1 (4%)	-	-
Severe hearing loss	3 (12%)	8 (32%)	-	-
Profound	4 (16%)	1 (4%)	-	-

**Figure 1:**
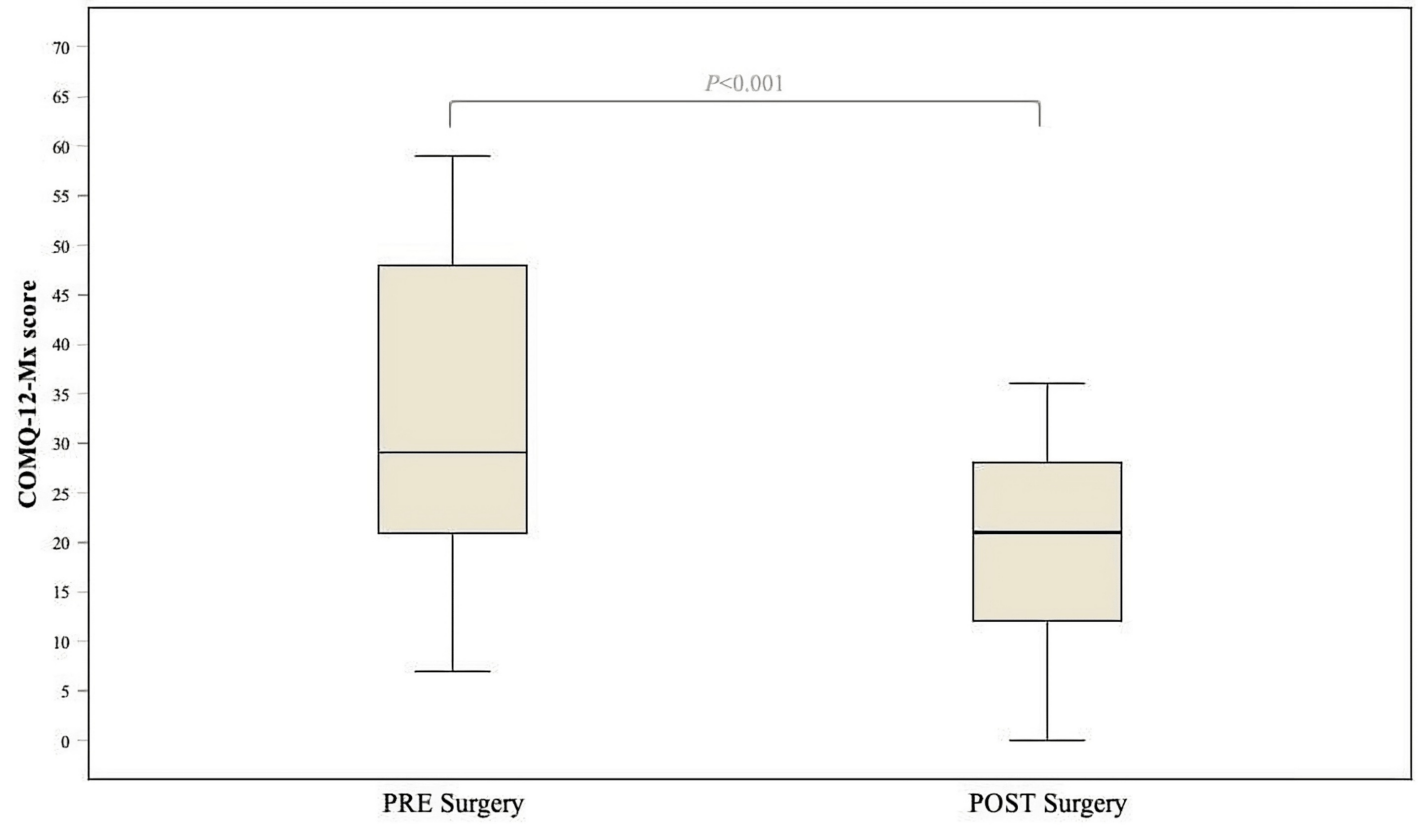
COMQ-12-Mx total scores Distribution of COMQ-12-Mx scores in 25 surgically treated patients. PRE denotes preoperative COMQ-12-Mx score; POST denotes postoperative COMQ-12-Mx score. Boxes display the interquartile range (25th-75th percentile); horizontal lines represent medians; whiskers extend to the minimum and maximum. Statistical analysis of paired groups was performed using the Wilcoxon signed-rank test (Z = -4.376, p < 0.001). A p-value <0.05 was considered statistically significant. Created by the authors; design inspired by Baetens et al. (2019) [11]. COMQ-12-Mx, Chronic Otitis Media Questionnaire-12, Mexican version

Although audiometric improvement did not reach statistical significance, analysis of the COMQ-12-Mx total score and all subdimensions revealed a significant postoperative decrease (Table 4), indicating an overall reduction in symptom severity and improvement in quality of life (Figure 1).

**Table 4:** Comparison of COMQ-12-Mx subdimension and total scores pre- and postoperatively (N = 25) Data are presented as median (IQR). ^a^p-value has been calculated using the Wilcoxon signed-rank test. A p-value < 0.05 was considered statistically significant. IQR, interquartile range; COMQ-12-Mx, Chronic Otitis Media Questionnaire-12, Mexican version

	Preoperative score	Postoperative score	Test value	p-value^a^
Symptom severity	18 (10.5 - 26.5)	9 (5 - 12.5)	-4.189	<0.001
Lifestyle and work impact	6 (1.5 - 9)	4 (0 - 7)	-2.055	0.040
Health services impact	6 (2.5 - 8.5)	3 (2 - 4.5)	-3.085	0.002
Emotional distress	4 (2.5 - 5)	3 (1 - 4)	-2.770	0.006
COMQ-12-Mx total score	29 (20.5 - 48.5)	21 (11 - 28)	-4.376	<0.001

Based on the Wilcoxon signed-rank test, the effect size was calculated for each individual question and for the total score (Table 5). A large effect was observed for the total COMQ-12-Mx score and for most individual items related to otorrhea, foul odor, hearing at home and in noisy environments, discomfort, vertigo, tinnitus, medical visits, medication use, and the patient’s emotional state, indicating a substantial improvement after surgery. Only two items - those assessing the impact on lifestyle activities and work performance - showed a medium effect, reflecting a moderate improvement following surgery.

**Table 5:** Effect size of each COMQ-12-Mx item ^a^p-value has been calculated using the Wilcoxon signed-rank test. A p-value < 0.05 was considered statistically significant. Created by the authors; design inspired by Baetens et al. (2019) [11]. COMQ-12-Mx, Chronic Otitis Media Questionnaire-12, Mexican version

	p-value^a^	Test value	R	Effect size
Question 1	<0.001	3.648	0.72	Large
Question 2	0.002	3.130	0.62	Large
Question 3	<0.001	3.589	0.71	Large
Question 4	0.005	2.784	0.55	Large
Question 5	0.001	3.271	0.65	Large
Question 6	0.002	3.061	0.61	Large
Question 7	0.002	3.032	0.60	Large
Question 8	0.051	2.000	0.39	Medium
Question 9	0.092	1.687	0.33	Medium
Question 10	0.007	2.701	0.54	Large
Question 11	0.010	2.580	0.51	Large
Question 12	0.006	2.770	0.55	Large
Total score	<0.001	4.376	0.87	Large

Intragroup analysis demonstrated improvement in the scores of most subdimensions and in the overall COMQ-12-Mx score for both the CWU and endoscopic techniques (Table 6).

**Table 6:** Comparison of COMQ-12-Mx item and subdimensions scores according to surgical technique (N = 25) Δ, difference between preoperative and postoperative scores. ^a^p-values calculated using the Wilcoxon signed-rank test, as described in the Statistical Analysis section. Due to the small sample size (n = 2), the CWD group was analyzed descriptively only. Statistical significance was set at p < 0.05. CWU: canal wall up; CWD: canal wall down; Endoscopic: endoscopic and retrograde techniques; PTA: pure-tone average; COMQ-12-Mx, Chronic Otitis Media Questionnaire-12, Mexican version

	Surgical technique	Preoperative score	Postoperative score		Δ (Preoperative - Postoperative)	Test value	p-value^a^
Symptom severity	CWU	18 (9.5 - 28)	9 (5 - 14.5)	5 (3 - 14.5)		-3.554	<0.001
	CWD	15.5	5	1.5		-	-
	Endoscopic	22.5 (9.5 - 28)	9 (3.75 - 11.5)	13.5 (6.25 - 17.75)		-2.207	0.027
Lifestyle impact	CWU	6 (0.5 - 9)	4 (0 - 5.5)	0 (0 - 2)		-1.428	0.153
	CWD	5	0	5		-	-
	Endoscopic	7.5 (3 - 10)	6.5 (0 - 9.25)	1 (-1.5 - 5.25)		-0.816	0.414
Health services impact	CWU	5 (2.5 - 9)	3 (2 - 4.5)	1 (0 - 16)		-2.502	0.012
	CWD	4	2.5	1.5		-	-
	Endoscopic	6 (4 - 8.5)	3.5 (0.75 - 7.5)	1 (0.25 - 5.25)		-1.511	0.131
Emotional distress	CWU	4 (0.5 - 4.5)	2 (0.5 - 4)	0 (0 - 1.5)		-1.992	0.046
	CWD	5	4	1		-	-
	Endoscopic	3.5 (2.75 - 5)	2.5 (0.75 - 4.25)	1.5 (0.25 - 2.25)		-1.633	0.102
COMQ-12-Mx total score	CWU	29 (16 - 49.5)	18 (10 - 28.5)	8 (3.5 - 22.5)		-3.624	<0.001
	CDW	29.5	20.5	9		-	-
	Endoscopic	33 (24.5 - 50)	22.5 (10.5 - 26.5)	16.5 (8.5 - 25)		-2.207	0.027
PTA, dB	CWU	55 (45 - 83.0)	53 (40 - 80.0)	3 (-0.5 - 14.5)		-2.048	0.041
	CWD	81.5	91.5	-10		-	-
	Endoscopic	37 (32.5 - 45.25)	34.5 (29.5 - 41.75)	3 (-2.5 - 7)		-1.156	0.248

In the CWU group, significant reductions were observed in symptom severity (preoperative 18 (9.5-28) vs postoperative 9 (5-14.5), p < 0.001), health services impact (preoperative 5 (2.5-9) vs postoperative 3 (2-4.5), p = 0.012), emotional distress (preoperative 4 (0.5-4.5) vs postoperative 2 (0.5-4), p = 0.046), and in the total COMQ-12-Mx score (preoperative 29 (16-49.5) vs postoperative 18 (10-28.5), p < 0.001).

Similarly, the endoscopic group demonstrated a significant decrease in symptom severity (preoperative 22.5 (9.5-28) vs postoperative 9 (3.75-11.5), p = 0.027) and in the total COMQ-12-Mx score (preoperative 33 (24.5-50) vs postoperative 22.5 (10.5-26.5), p = 0.027), whereas the lifestyle impact and emotional distress domains showed non-significant improvements.

Audiological evaluation revealed a significant improvement in PTA within the CWU group (preoperative 55 dB (45-83) vs postoperative 53 dB (40-80), p = 0.041). No significant differences were observed in the endoscopic group.

The CWD group (n = 2) was analyzed descriptively due to the small sample size; however, both patients showed improvement across all COMQ-12-Mx domains and in the total score. The ΔPTA was -10.0 dB, indicating no postoperative recovery and instead suggesting a mild deterioration in hearing thresholds.

## Discussion

In the present study, the impact of surgical treatment on quality of life and hearing function was evaluated in patients with COMC using the COMQ-12-Mx questionnaire [9]. The results showed a significant postoperative improvement in quality of life, evidenced by the reduction in the total COMQ-12-Mx score from 29 to 21 points (p < 0.001), which is consistent with findings reported by various authors internationally [12]. Demirag Evman and Cakil [10] observed a decrease in the COMQ-12 score from 25 to 7 after type I tympanoplasty, reflecting symptomatic and functional improvement comparable to our findings. Similarly, Baetens et al. [11] reported a significant reduction in symptoms and daily limitations after tympanoplasty with bony obliteration, with up to 50% of patients reaching normal quality-of-life levels. Consistently, the systematic review by Schouwenaar et al. [12], which included 16 studies, confirmed a significant improvement in quality of life following otologic surgery, particularly when specific instruments such as the COMQ-12 are used, reinforcing the sensitivity of this tool for detecting clinically relevant changes.

Regarding auditory function, our study showed a trend toward improvement in PTA (from 48 to 42 dB) without reaching statistical significance (p = 0.059). This finding may be attributed to the heterogeneity of the surgical techniques employed and the variability in disease stage among patients. Previous studies, such as those by Lucidi et al. [13] and Maile et al. [16], also demonstrated that, although surgery generally improves quality of life, hearing recovery may vary depending on the type of intervention, the extent of the cholesteatoma, and the preservation of the ossicular chain. The profile of the included patients reflected an adult population (median age: 47 years) with a predominance of females (56%), similar to other series [7,10,13]. The presence of comorbidities, such as type 2 diabetes mellitus (32%) and obesity (12%), may influence wound healing and complication rates, which were observed in 24% of cases, mainly involving neotympanum perforation and graft failure. These results are consistent with previous reports describing postoperative complication rates between 15% and 25% [5,7,13].

The significant improvement in quality of life observed three months after surgery confirms that the procedure not only corrects the structural disease but also favorably impacts psychological, emotional, and social aspects, as described by Baetens et al. [11] and Maile et al. [16]. This underscores the importance of incorporating tools such as the COMQ-12-Mx into the comprehensive evaluation of patients with COMC, extending beyond traditional audiometric parameters.

This study has limitations. First, the single-group pre-post design without a parallel control group limits the ability to distinguish surgical effects from natural symptom variation; accordingly, the observational design supports association rather than causation. Second, although the calculated sample size for the primary outcome was achieved, the overall cohort remains relatively small, reducing the statistical power of within-technique subgroup analyses; therefore, these analyses were considered exploratory and should be interpreted with caution. Third, we did not adjust for potential confounders, such as comorbidities, which may influence postoperative healing or patient outcomes. Fourth, the short follow-up period (three months) does not allow assessment of long-term hearing stability, complications, and quality-of-life outcomes. The presence of bilateral disease in some patients could have introduced bias in the overall perception of impact, although it was minimized through standardized methodology. Finally, being a single-center study, the generalizability of the findings is limited. Despite these limitations, the prospective design, systematic measurements, and the consistency of the results with international literature support the clinical validity and applicability of the findings to otologic practice.

## Conclusions

In this study, surgery for COMC resulted in a significant within-group postoperative improvement in disease-related quality of life, particularly in symptom burden and emotional impact. Although both surgical approaches demonstrated postoperative benefit, the small sample size does not allow determination of their comparative effectiveness. Hearing outcomes varied across techniques; however, the overall trend toward improvement reinforces the therapeutic value of surgical management. The COMQ-12-Mx reliably detected postoperative changes and proved to be a valuable patient-reported outcome measure for assessing clinical and functional results following otologic surgery.
